# Carbolic Acid in Indigestion

**Published:** 1885-06-20

**Authors:** 


**Carbolic Acid in Indigestion.**—Berdoe frequently treated acid dyspepsia with small doses of carbolic acid. He uses a solution suggested by Dr. Fenwick, containing one part of the crystallized acid in four parts of glycerin, and gives from five to ten minims as a dose, either merely diluted or mixed with nux vomica or liquor opii sedativus, the latter being added in case of pain. He does not attempt to explain the action of the drug, but suggests that its efficacy may be due either to its anti-fermentative power, or to the anæsthesia which it induces in the gastric mucous membrane.—*British Medical Journal.*

